# Single-Dose Intranasal Immunisation with Novel Chimeric H1N1 Expressing the Receptor-Binding Domain of SARS-CoV-2 Induces Robust Mucosal Immunity, Tissue-Resident Memory T Cells, and Heterologous Protection in Mice

**DOI:** 10.3390/vaccines11091453

**Published:** 2023-09-04

**Authors:** Donghong Wang, Yao Deng, Jianfang Zhou, Wen Wang, Baoying Huang, Wenling Wang, Lan Wei, Jiao Ren, Ruiwen Han, Jialuo Bing, Chengcheng Zhai, Xiaoyan Guo, Wenjie Tan

**Affiliations:** 1 Key Laboratory of Biosafety, National Health Commissions, National Institute for Viral Disease Control and Prevention, China CDC, 155 Changbai Road, Beijing 102206, China; dhwang666@163.com (D.W.);; 2 State Key Laboratory for Molecular Virology and Genetic Engineering, Chinese National Influenza Center, National Institute for Viral Disease Control and Prevention, China CDC, 155 Changbai Road, Beijing 102206, China; 3 Zhejiang Provincial Key Laboratory of Medical Genetics, School of Laboratory Medicine and Life Sciences, Wenzhou Medical University, Wenzhou 325035, China; 4 School of Public Health, Xinxiang Medical University, Xinxiang 453003, China

**Keywords:** COVID-19, influenza virus, heterologous protection, recombinant vaccine, T cell immunity

## Abstract

Current COVID-19 vaccines can effectively reduce disease severity and hospitalisation; however, they are not considerably effective in preventing infection and transmission. In this context, mucosal vaccines are pertinent to prevent SARS-CoV-2 infection and spread. In this study, we generated a replication-competent recombinant chimeric influenza A virus (IAV) expressing the receptor-binding domain (RBD) of a SARS-CoV-2 prototype in the C-terminus of the neuraminidase (NA) of A/Puerto Rico/08/1934 H1N1 (PR8). The remaining seven segments from A/WSN/1933 H1N1 (WSN) were named PR8NARBD/WSN. We observed that the recombinant virus with the WSN backbone demonstrated improved expression of NA and RBD. A single intranasal dose of PR8NARBD/WSN(10^3^PFU) in mice generated robust mucosal immunity, neutralising antibodies, cellular immunity, and tissue-resident memory T cells specific to SARS-CoV-2 and IAV. Importantly, immunisation with PR8NARBD/WSN viruses effectively protected mice against lethal challenges with H1N1, H3N2 IAV, and SARS-CoV-2 Beta variant and significantly reduced lung viral loads. Overall, our research demonstrates the promising potential of PR8NARBD/WSN as an attractive vaccine against emerging SARS-CoV-2 variants and influenza A virus infections.

## 1. Introduction

Coronavirus disease 2019 (COVID-19), caused by the severe acute respiratory syndrome coronavirus 2 (SARS-CoV-2), is a severe disease that results in widespread morbidity and mortality worldwide [1]. As of 1 June 2023, there were 767 million confirmed cases of COVID-19 worldwide, of which 6.9 million patients died of viral infections or other related complications (https://covid19.who.int/). Over a short period of time, several vaccine platforms have been developed globally and used to immunise the majority of individuals after ensuring their safety and effectiveness. Most COVID-19 vaccines are intramuscular injections, which can induce both humoral and cellular immune responses to reduce disease severity, hospitalisation, and mortality [2,3,4]. However, emerging SARS-CoV-2 variants threaten the effectiveness of the current COVID-19 vaccines. Frequent outbreaks of SARS-CoV-2 infections emphasise the necessity of alternative vaccination approaches with the potential to enhance upper respiratory immunity [5,6].

The influenza A virus (IAV) vector shows great potential as a platform for vaccine development because of its ability to elicit strong mucosal, humoral, and cellular immunity and to be administered in a needle-free manner at mucosal sites [7,8]. The IAV vector has been successfully used to express various pathogens [9,10,11], with most foreign genes being inserted into the haemagglutinin (HA), neuraminidase (NA), or non-structural (NS) genes of the IAV [8]. To date, several IAV vector-based vaccines against SARS-CoV-2 have been developed. However, most of these vaccines require a prime-boost immunisation regime [7,12,13,14], which is impractical and difficult to implement in large-scale vaccination programs. Single-dose intranasal immunisation with influenza virus vector COVID-19 vaccine has previously been reported, which only evaluated humoral immunity in immunised mice, and did not examine mucosal and cellular immunity [15]. Therefore, further research must be conducted to develop single-dose vaccines. Furthermore, not all IAV vector-based vaccines provide cross-protection against different IAV subtypes, making it essential to develop vaccines that offer broad protection against a vast range of IAVs. Finally, although various IAV vector-based vaccines have shown promising results in inducing strong immune responses, limited information is available on the generation and persistence of memory T cells after vaccination. Therefore, further studies are warranted to better understand the memory T cell response elicited by these vaccines and their potential contribution to achieving heterologous protection against the virus.

Research on a single dose of an IAV-based SARS-CoV-2 vaccine that can produce not only humoral immunity, but also mucosal, cellular, and memory T cell immunity against both influenza virus and SARS-CoV-2 is lacking. The vaccine candidate developed in this study may produce protective immunity against challenges with heterogeneous IAVs and SARS-CoV-2, implying its promising potential as bivalent vaccine for influenza and COVID-19.

## 2. Materials and Methods

### 2.1. Cell Lines

Human embryonic kidney (HEK293T; ATCC) and Madin-Darby canine kidney (MDCK; ATCC) cells were maintained in Dulbecco’s minimal essential medium (Gibco^TM^) supplemented with 10% foetal bovine serum (PAN, Cat:P30-3302) and 1% penicillin-streptomycin (PS, Cat:15140-122).

### 2.2. Construction of Plasmids and Generation of Recombinant Viruses

The chimeric A/Puerto Rico/08/1934 H1N1 (PR8) NA incorporated with the RBD of SARS-CoV-2 (Wuhan-Hu-1, [Genbank:MN908947], residues 331-531) was linked to via the porcine teschovirus (PTV)-1 2A autocleavage peptide sequence ‘GSGATNFSLLKQAGDVEENPGP’ and the Gaussia luciferase signal peptide, retaining the 3′ and 5′ packaging signals of the NA. The eight A/WSN/1933 (WSN) plasmids were synthesised using Genscript, with the GenBank number published in a previous study [16] and cloned into the plasmid pHW2000. The recombinant viruses were rescued using the pHW2000-based eight-plasmid system described by Hoffmann et al. [17]. Briefly, co-culture of 293T/MDCK cells were co-transfected with a PR8NARBD/pHW2000 plasmid and the other seven plasmids of PR8 (HA, M, NS, PB1, PB2, NP and PA) or WSN incubated overnight at 37 °C with an added 2 μg/mL of tosylsulfonyl phenylalanyl chloromethyl ketone (TPCK)-treated trypsin (Sigma, USA). After 48 h, the supernatant was harvested as passage 0 (P_0_) virus and subsequently passaged into 9-day-old embryonated chicken eggs. After 48 h inoculation, the rescue of recombinant viruses was confirmed by hemagglutination of chicken red blood cells. The virus titres were determined using TCID50 and calculated by the Reed and Muench method.

### 2.3. Animal Challenge and Evaluation of Protection

All 6–8-week-old BALB/c mice allowed free access to water and diet and provided with a 12 h light/dark cycle (temperature: 18–28 °C, humidity: 40–70%). The mice were bred and maintained in a specific-pathogen-free (SPF) environment at the Laboratory Animal Center of Sinovac Co., Ltd, China. For the IAV challenge, the BALB/c mice were purchased from Beijing Vital River Laboratory Animal Technology Co., Ltd., China, and underwent immunisation with either 10^2^PFU, 10^3^PFU PR8NARBD/WSN, or 10^3^PFU PR8NA/WSN viruses *(n* = 8/group). They were anesthetised with 1% pentobarbital sodium and intranasally challenged with a reassortant virus containing 3LD50 (50% tissue lethal dose) of A/Puerto Rico/08/1934 H1N1, rgA/X31/H3N2 (with HA and NA gene segments from X31/H3N2, and the remaining six genome segments from PR8) [18], and rgA/Anhui/H7N9 (with HA and NA gene segments from AnHui/H7N9, and the other six genome segments from PR8) 28 days after a single intranasal vaccination [19]. After virus challenge, the body weight of each mouse was measured daily, and their fur condition and behavioural state were monitored. Mice that lost 25% of initial body weight were scored as dead and euthanised (https://www.humane-endpoints.info/en).

The SARS-CoV-2 challenge assay was conducted in an Animal Biosafety Level 3 laboratory. The SARS-CoV-2 Beta virus strains of 501Y.V2 were used [20]. Virus titre was determined using a micro-cytopathogenic efficiency (CPE) assay on Vero cells. The Beta strain was subjected to serial 10-fold dilutions and used to infect Vero cells. After incubating for 4 days in a 5% CO_2_ incubator at 37 °C, the cells were examined under the microscope to observe cytopathic effects. The virus titre was calculated using the Karber method [21]. BALB/c mice were challenged with SARS-CoV-2 Beta strains. Briefly, the mice (*n* = 5) were directly infected with 1 × 10^5^ TCID50 of SARS-CoV-2 Beta variable strain. Vaccine-induced protection in mice was evaluated as described previously on day 4 post-challenge.

### 2.4. Enzyme Linked Immunosorbent Assay

An enzyme-linked immunosorbent assay (ELISA) was used to measure the RBD, and H1 HA-binding IgG antibodies and secretory IgA in the bronchoalveolar lavage fluid (BAL) were detected as previously described [7]. In brief, a 96 well EIA/RIA plate (Corning Inc., USA, Corning) was coated with 100 ng/well RBD protein of SARS-CoV-2 (Sino biological, China, cat:40592) and influenza virus HA protein (Sino biological, China, cat:11684-V08B). After blocking with 10% goat serum, serially twice-diluted sera was plated into each well and incubated at 37 °C for 2 h. This was followed by washing with PBST (0.05%Tween-20). For IgG antibody detection, incubated with HRP-conjugated goat anti-mouse (1:5000) at 37 °C for 1 h. For the detection of sIgA, anti-mouse IgA mAb MT39A (Mabtech, Swedish, cat:3885-6-100) (1:1000) serves as the secondary antibody for detecting sIgA, incubated with streptavidin-HRP (1:1000) at 37 °C for 1 h. The plates were developed with tetramethylbenzidine (TMB) followed by the addition of 2 M H_2_SO_4_ to stop the reaction and read at 450 nm. The serum-binding antibody titre was defined as the highest dilution that yields an absorbance of 2-fold greater than background value. Data analysis was conducted using GraphPad Prism 8.0.

### 2.5. Interferon-γ ELISpot Assay

Interferon-γ (IFN-γ) ELISpot assays were performed according to the BD^TM^ ELISpot kit’s protocol. Synthetic RBD peptides of SARS-CoV-2 prototype strain and nucleoprotein (NP)-positive peptides of H1N1 (NP1415: RLIQNSLTIERMVLS; NP1385: TYQRTRALV) were used. The spots were scanned and quantified using an ImmunoSpot CTL reader. The spot-forming units (SFUs) per million cells were calculated by subtracting the number of negative control wells.

### 2.6. Intracellular Cytokine Staining

Lung samples were collected 14 days after vaccination. Single-cell suspensions were digested with 1 mg/mL collagenase (Roche, Swedish, cat:11088882001) and 100 µg/mL DNase I (Roche, cat:110104159001). A total of 2 × 10^6^ cells were used and restimulated with the RBD peptides (10 µg/mL) of prototype SARS-CoV-2 strain or the NP peptides (5 µg/mL) of H1N1. On the second day, the cells were stained dead or live with ZombieAqua (BioLegend, USA, cat:423101) at room temperature for 20 min in the dark. Cells were blocked with an Fc receptor anti-mouse CD16/32 antibody (clone93, BioLegend) for 10 min on ice. Thereafter, pneumonocytes were stained with the following surface antibodies: anti-CD4 (clone:RM4-5, Biolegend), anti-CD8α(clone:53-6.7, Biolegend), anti-CD44 (clone:IM7, Biolegend), anti-CD62L (clone:MEL-14,Biolegend), anti-CD103 (clone:2E7, Biolegend), and anti-CD69 (clone:H1.2F3, Biolegend). Cells were incubated for 30 min in the dark for the surface staining. The samples were fixed, permeabilised (Cytofix/Cytoperm, USA, cat:554714), and intracellularly stained with IFN-γ(clone XMG1.2, BioLegend). Data were collected using FACS II and analysed using FlowJo V10.

### 2.7. Histopathology Analysis

Mouse lung tissue was fixed with 4% paraformaldehyde and embedded with paraffin. Haematoxylin and eosin-stained sections were prepared for pathological evaluation according to the International Harmonisation of Nomenclature and Diagnostic Criteria (INHAND) scores. Tissue sections 4 µm thick were stained with haematoxylin and eosin (H&E). The samples were rated by the following lung histological scores: 0 (no obvious pathological changes); 1 (minimum, 0~20% of tissues were affected by the lesion); 2 (mild, 21~40% of tissues are affected by the lesion); 3 (moderate, 41~60% of tissues were affected by the lesion); 4 (moderately severe, 61~80% of tissues were affected by the lesions); 5 (severe, 81~100% of tissues were affected by the lesion).

### 2.8. Statistical Analyses

All statistical analyses were performed using the GraphPad Prism software. One-way analysis of variance (ANOVA) or *t*-tests were used to determine the statistical significance with means ± standard error of means (SEM).

## 3. Results

### 3.1. Generation of Replication-Complement IAV Carrying the RBD of SARS-CoV-2 with A/Puerto Rico/08/1934 H1N1 and A/WSN/1933 Backbones

**Figure 1:**
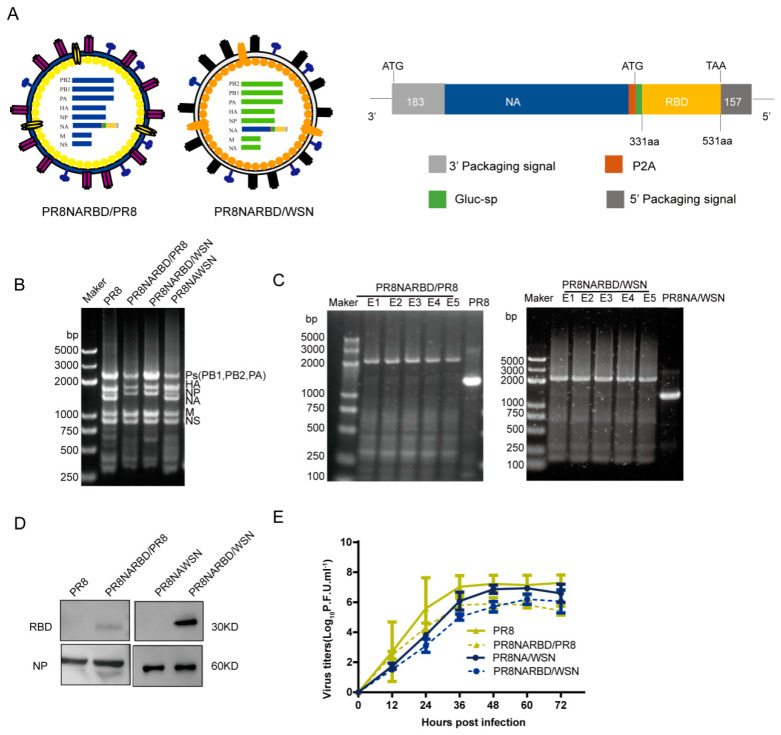
Generation and characterisation of replication-complement influenza virus carrying the RBD of SARS-CoV-2 with A/Puerto Rico/08/1934 H1N1 (PR8) and A/WSN/1933 (WSN) backbone, respectively. (**A**) Diagram representation of the recombinant influenza viruses carrying the RBD of SARS-CoV-2 in the PR8 NA genomics segment with PR8 or WSN backbone, respectively. Each element is shown. (**B**) Influenza virus whole genome amplification. (**C**) Genetic stability of recombination PR8NARBD/PR8 and PR8NARBD/WSN. (**D**) Immunoblot analysis of RBD and NP expression in concentrated recombination viruses amplified in embryonated eggs. (**E**) Growth kinetics of recombination virus on Madin-Darby canine kidney (MDCK) cells compared to wild-type.

Previous studies have shown that the WSN virus expresses more NA than that of the PR8 virus [16]. In this study, replication-competent recombinant IAVs carrying the RBD of SARS-CoV-2 in the PR8 NA segment were rescued using seven gene segments from PR8 and WSN, named PR8NARBD/PR8 and PR8NARBD/WSN. PR8 virus and PR8NAWSN virus were rescued in parallel (Figure 1A).

To evaluate the presence of recombinant IAV-fused RBD in the C-terminus of NA, the rescued recombinant viruses were verified by simultaneously amplifying eight genomic RNA segments using RT-PCR. The chimeric NA gene segment band size increased from 1400 to 2300 bp, which represented the range of polymerase gene fragments (PB1, PB2, and PA) on agarose gel electrophoresis (Figure 1B). The stability of the chimeric NARBD genes was assessed in embryonated chicken eggs. The RBD gene was preserved in the chimeric NA vRNA for at least five passages (Figure 1C). We showed that RBD protein expression in the WSN backbone was higher than that in the PR8 backbone; NP protein was used as a control for virions (Figure 1D) (details regarding the WB experiment can be found in Supplementary Methods S1.2). The growth kinetics of the recombinant PR8NARBD/WSN and PR8NARBD/PR8 viruses were approximately equivalent to those of the PR8NAWSN and PR8 viruses in MDCK cells (Figure 1E). No mutations were detected in the RBD gene.

### 3.2. Recombinant PR8NARBD/WSN Virus Demonstrates Higher RBD and NA Expression Levels and Greater Attenuation than PR8NARBD/PR8 Virus In Vitro

**Figure 2:**
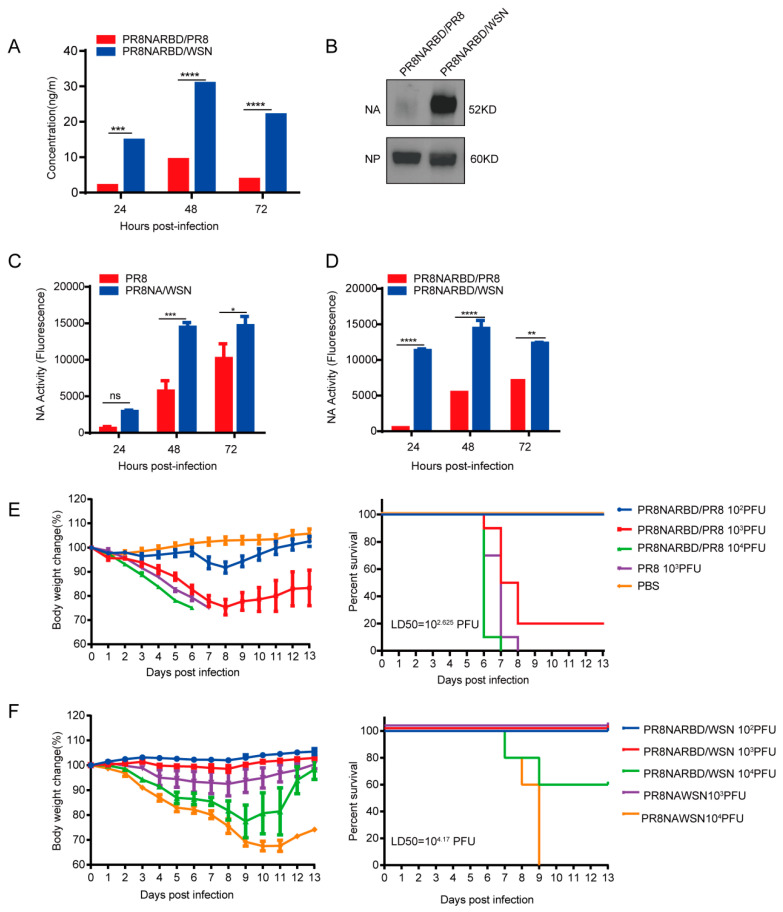
The RBD and NA expression levels of PR8NARBD/PR8 and PR8NARBD/WSN viruses in vitro and their pathogenicity in mice were detected. (**A**) The recombination viruses infected supernatants were collected every 24 h and detected the RBD concentration. (**B**) Immunoblot analysis of NA expression in the purified virions (0.5 μg per lane) by sucrose gradient. (**C**,**D**) The infected supernatants were collected every 24 h and detected the NA activity with the MUNANA substrate. (**E**) Pathogenicity and survival of recombinant influenza viruses with PR8 backbone. (**F**) Pathogenicity and survival of recombinant influenza viruses with WSN backbone. The body weight was monitored for fourteen days. Mice that lost 25% of initial body weight were scored as dead and euthanised. Statistical significance was analysed by two-way analysis of variance (ANOVA). The bars plotted show means ± SEM. The results represent three independent experiments. (* *p* < 0.05, ** *p* < 0.01, *** *p* < 0.001, **** *p* < 0.0001; *ns*, *no statistical difference*).

The RBD and NA expression levels in recombinant PR8NARBD/WSN and PR8NARBD/PR8 viruses were compared. RBD-specific ELISAs and NA activity assays were performed to detect RBD expression in the infected supernatants (details regarding the NA activity and RBD ELISA experiment can be found in Supplementary Methods S1.3 and S1.4). The results showed that recombinant viruses with the WSN backbone had higher RBD expression (3~7 fold) (Figure 2A) and NA activity (2~5 fold) than those with the PR8 backbone (Figure 2C,D).

We further characterised NA protein expression in vitro. Embryonated eggs were inoculated with 100 PFU recombinant virus, the allantoic fluid was collected and purified using a sucrose gradient, and 0.5 μg of the purified protein was analysed using Western blotting. The results indicated a significantly higher NA protein expression in the WSN backbone than in the PR8 backbone (Figure 2B).

We assessed whether insertion of the RBD affects viral virulence. Six eight-week-old female BALB/c mice (*n* = 5 for each group) received a single intranasal immunisation of PR8NARBD/PR8 and PR8NARBD/WSN at a 10-fold rate of increase from 10^2^ PFU to 10^4^ PFU per mouse or PR8 and PR8NAWSN at 10^3^PFU per mouse. Mice infected with 10^3^ PFU of the PR8NARBD/PR8 virus had a higher survival rate than those infected with 10^3^ PFU of the PR8 virus (Figure 2E). For the WSN backbone, mice infected with 10^4^PFU of PR8NARBD/WSN experienced a lower weight decrease and a higher survival rate (60%) than those infected with 10^4^PFU of PR8NAWSN, resulting in a rapid weight decrease and death within 9 days (Figure 2F). Overall, these results suggest that the insertion of the RBD reduced viral virulence in mice, irrespective of the backbone used. Importantly, virus with the WSN backbone demonstrated more evident attenuation than that with the PR8 backbone. The 50% tissue lethal dose (LD50) of PR8NARBD/WSN (LD50 = 10^4.17^ PFU) was 35 times lower than that of PR8NARBD/PR8 (LD50 = 10^2.625^ PFU) in mice (Figure 2E,F). These results suggest that chimeric PR8NARBD/WSN significantly attenuates virulence in mice and may be a promising IAV vector.

### 3.3. Single Intranasal Immunisation with PR8NARBD/WSN Induces Robust Humoral and Mucosal Immunity

**Figure 3:**
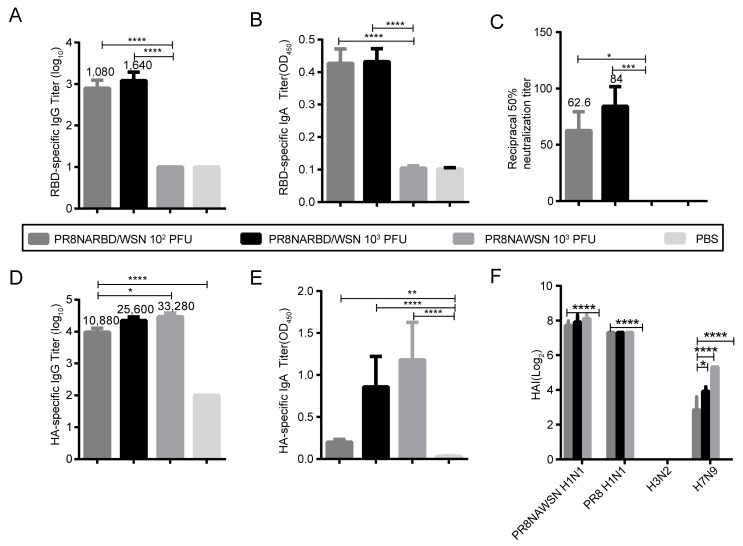
Humoral immune and mucosal immune responses elicited by single-dose intranasal immune PR8NARBD/WSN viruses in BALB/c mice. (**A**) RBD-specific IgG antibodies. (**B**) RBD-specific IgA antibodies in the BALF. (**C**) RBD-specific neutralisation antibody. (**D**) HA-specific IgG antibodies. (**E**) HA-specific IgA antibodies in the BALF (1:10 dilution). (**F**) Serum HAI titres against the PR8NAWSN virus, A/Puerto Rico/08/1934 H1N1 (PR8) virus, A/X31/H3N2 virus, A/Anhui/H7N9 virus. Statistical significances were analysed by one-way analysis of variance (ANOVA). The bars plotted show means ± SEM. The results represent three independent experiments. (* *p* < 0.05, ** *p* < 0.01, *** *p* < 0.001, **** *p* < 0.0001).

We evaluated whether the PR8NARBD/WSN virus triggers humoral and mucosal immunity against SARS-CoV-2 and IAV in vivo. BABL/c mice were immunised intranasally with 10^2^PFU and 10^3^PFU of PR8NARBD/WSN as well as 10^3^PFU of PR8NAWSN. Serum and bronchoalveolar lavage (BAL) samples were collected at 14 days after one dose of immunity (*n* = 5 per group). The results showed that a single-dose immunisation with the PR8NARBD/WSN virus could induce the production of RBD-specific binding antibodies (Figure 3A), mucosal immunity (Figure 3B) and neutralising antibodies (Figure 3C) [22] and that the PR8NARBD/WSN virus could induce the production of HA-specific binding antibodies (Figure 3D) and mucosal immunity against HA in mice (1:10 dilution) (Figure 3E). Haemagglutination inhibition (HAI) assays were used to measure the HAI activity of sera from mice immunised against various IAVs. The vaccination group produced high levels of antibodies against the PR8NAWSN H1N1 and PR8 H1N1 viruses and relatively low levels of antibodies against the H7N9 virus. No antibodies against H3N2 were detected (Figure 3F).

Notably, no antibodies against the RBD were detected in the group immunised with 10^2^PFU PR8NARBD/PR8 (Supplementary Figure S1A,B). However, both PR8NARBD/WSN and PR8NARBD/PR8 viruses were still able to induce comparable levels of HAI against IAV (Supplementary Figure S1C). Furthermore, the PR8NARBD/WSN virus induced higher levels of RBD antibody expression in mice than the PR8NARBD/PR8 virus. Overall, these findings suggest that PR8NARBD/WSN is effective at eliciting both systemic and mucosal immunity against SARS-CoV-2 and IAV, making it a potential candidate for vaccine development.

### 3.4. Single-Dose Intranasal Immunisation with PR8NARBD/WSN Virus Induces Significant Levels of Both Cell-Mediated Immune Responses and Tissue-Resident Memory T Cells

**Figure 4:**
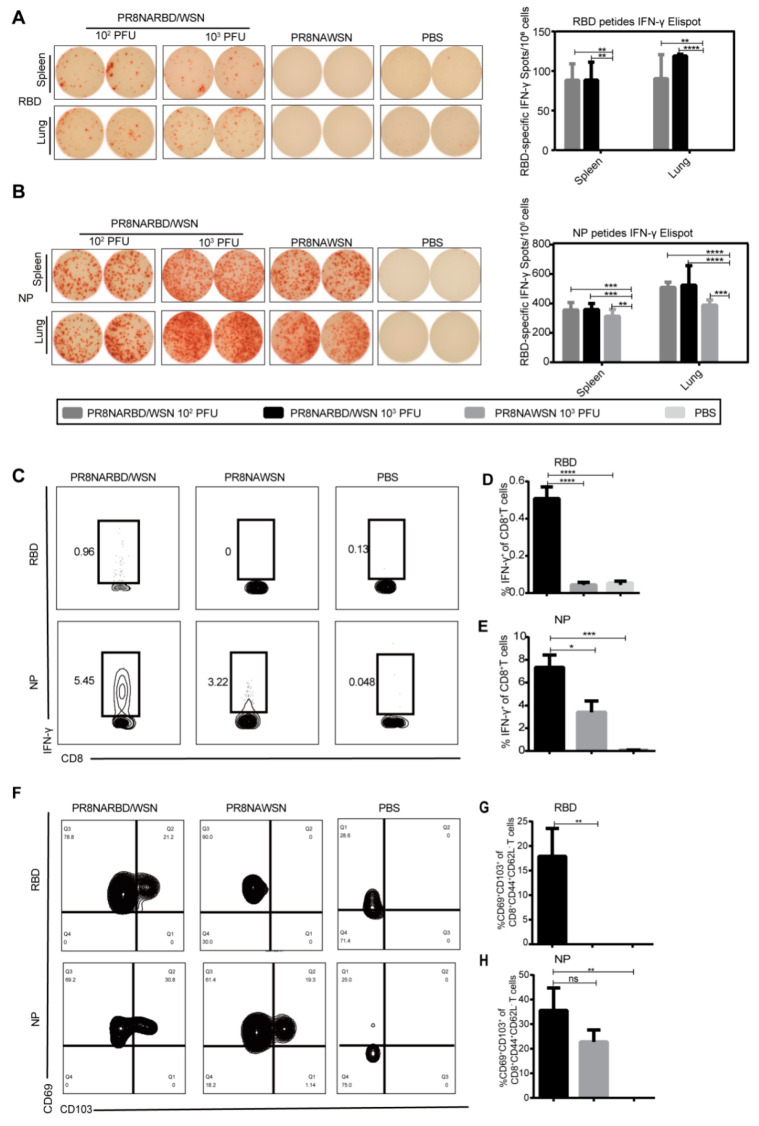
Cell-mediated immune responses and tissue-resident memory T cell were detected by ELISpot assay and flow cytometry assay. (**A**,**B**) Splenocytes and pneumonocytes were obtained on day 14 after a single intranasal administration of PR8NARBD/WSN and evaluated cellular responses by ELISpot assay. (**C**) Representative flow cytometry plots for IFN-γ expression by CD8^+^ cells. Pneumonocytes were stimulated with RBD peptide and NP peptide, which was assessed by flow cytometry assay. (**D**,**E**) Percentages of RBD- or NP-specific IFN-γ-positive T cells among total CD8^+^ T cell populations in the lungs. (**F**) Expression of CD69 and CD103 markers by SARS-CoV-2 or IAV-specific CD8 tissue resident memory (Trm) cells induced by immunisation with PR8NARBD/WSN. (**G**) Percentages of RBD-specific CD69^+^CD103^+^ CD8 Trm cells. (**H**) Percentages of NP-specific CD69^+^CD103^+^CD8 Trm cells. Data were analysed with one-way ANOVA with means ± SEM. (* *p* < 0.05, ** *p* < 0.01, *** *p* < 0.001, **** *p* < 0.0001; *ns*, *no statistical difference*).

T cells play a pivotal role in providing long-term heterologous protection against coronaviruses [23,24]. To test the T cell-mediated immune responses by single-dose intranasal immunisation with PR8NARBD/WSN virus in mice, splenocytes and pneumonocytes were obtained on day 14 and assessed using IFN-γ-specific ELISpot as well as intracellular staining using flow cytometry. The results showed a significant number of IFN-γ-secreting cells in the splenocytes and pneumonocytes after stimulation with RBD or NP peptide pools, indicating that a single dose of PR8NARBD/WSN elicited antigen-specific cell-mediated immune responses in splenic and pulmonary cells. Notably, the NP peptide-stimulated cellular immunity was significantly higher than the RBD peptide-stimulated cellular immunity (Figure 4A,B). However, cellular immunity against RBD was not detected in the group immunised with 10^2^PFU PR8NARBD/PR8, and NP-specific cellular immunity was detected at a level similar to that of PR8NARBDWSN (Supplementary Figure S1D,E).

We tested the RBD-specific CD8^+^T cell response in the lungs. Single-dose intranasal immunisation with the PR8NARBD/WSN virus induced an RBD-specific CD8^+^T cell response by flow cytometry (Figure 4C,D). In the PR8NARBDWSN- and PR8NAWSN-immunised groups, we observed a robust NP-specific CD8^+^T cell response in the lungs. (Figure 4C–E). Overall, the above data indicate that a single-dose intranasal immunisation with the PR8NARBD/WSN virus induces strong cellular immune responses to SARS-CoV-2 and IAV.

Tissue-resident memory T cells (Trm) play an important role in defending against SARS-CoV-2 and IAV and providing heterosubtypic immunity among respiratory viruses [25,26]. The co-expression of CD69 and CD103 is a signature marker of Trm cells and can be used to identify CD8^+^Trm in mouse lungs. After a single dose of intranasal immunisation with PR8NARBD/WSN, lung lymphocytes were collected 14 days later, and the expression of memory pneumonocytes was detected with RBD and NP peptides (Figure 4F) [26] and shown in Supplementary Figure S2. In the PR8NARBD/WSN- immunised group, 17.92% of RBD-specific CD8^+^ Trm cells (Figure 4G) and 35.6% of NP-specific CD8^+^ Trm cells (Figure 4H) in the lung lymphocytes expressed both CD69 and CD103 markers. These results suggest that a single intranasal dose of PR8NARBD/WSN induced the generation of memory cells specific to NP and RBD in the lungs.

### 3.5. Single-Dose Intranasal Immunisation with PR8NARBD/WSN Provides Heterologous Protection against Both IAV and SARS-CoV-2 Challenges

**Figure 5:**
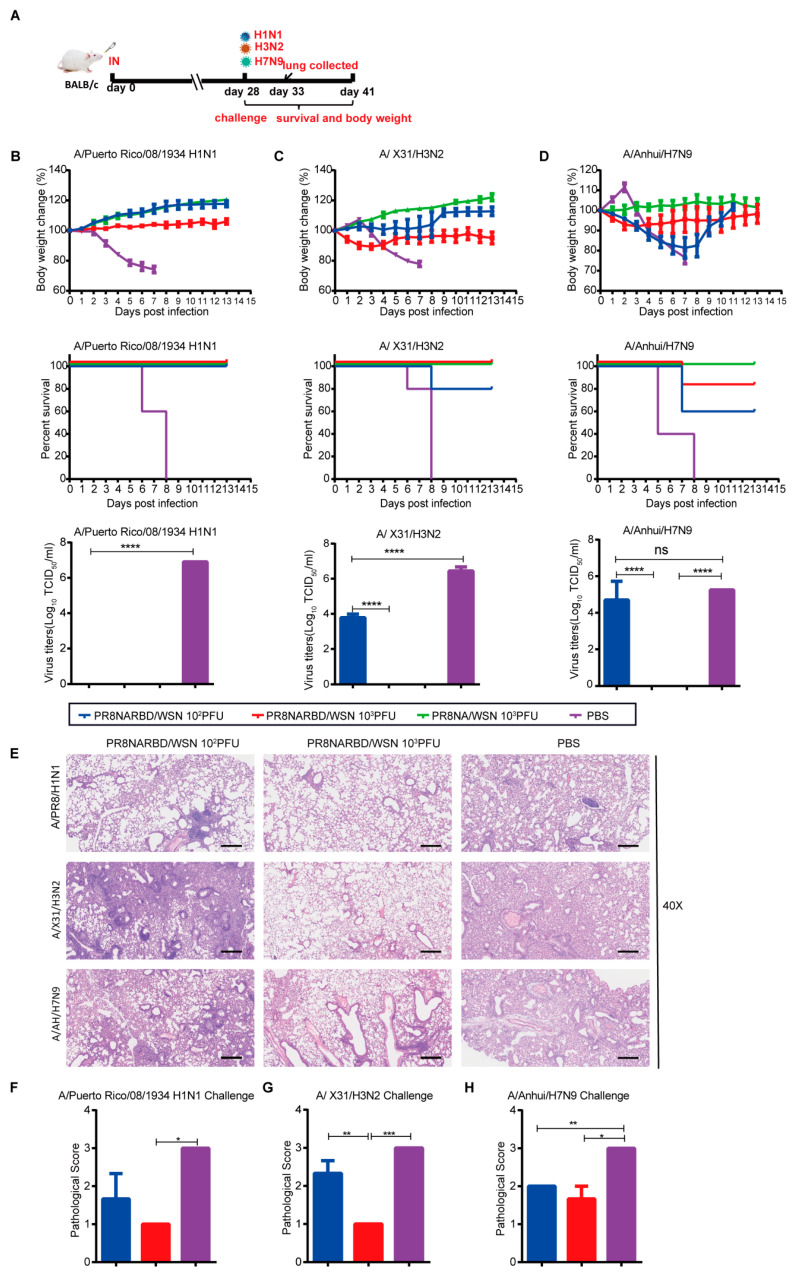
Single intranasal vaccination PR8NARBD/WSN protects mice from both homologous and heterologous influenza virus challenge. (**A**) Schematic of timeline for intranasal vaccination and influenza virus challenge. (**B**) Vaccinated mice were challenged with PR8 virus. PR8 titres in lungs were detected on day 5 post-challenge. (**C**) rgA/X31/H3N2 challenge at 28 days after one-dose vaccination. H3N2 titer in lung (**D**) rg A/Anhui/H7N9 virus challenge at 28 days after one-dose vaccination. H7N9 titre in lung. Body weights and survival were observed for 14 days *(n* = 5 mice per group). (**E**) Histopathological examinations of lungs from vaccinated mice collected on day 5 after challenge with influenza viruses (*n* = 3). Scale bar, 100 μm. (**F**–**H**) Comprehensive pathological score of mouse lung. Blue represents PR8NARBD/WSN 10^2^PFU, red represents PR8NARBD/WSN 10^3^PFU, and purple represents the PBS group. Data were analysed with one-way ANOVA. Error bars represent mean values ± SEM (* *p* < 0.05, ** *p* < 0.01, *** *p* < 0.001, **** *p* < 0.0001; *ns*, *no statistical difference*).

To evaluate the protective effects of PR8NARBD/WSN against IAV, mice were intranasally challenged with H1N1 (A/Puerto Rico/08/1934), rgH3N2 (A/X31/H3N2), or rgA/Anhui/H7N9 virus 28 days after a single-dose intranasal vaccination (Figure 5A). Immunisation with either 10^2^PFU, 10^3^PFU PR8NARBD/WSN, or 10^3^PFU PR8NA/WSN viruses offered full protection against lethal challenge with the PR8 H1N1 virus, with no weight loss and 100% survival (Figure 5B). No virus was detected in the lung tissues of vaccinated mice (Figure 5B). Furthermore, the 10^3^PFU PR8NARBD/WSN and 10^3^PFU PR8NAWSN groups showed good protection against H3N2 viruses, with no weight loss and 100% survival. However, immunisation with the 10^2^PFU PR8NARBD/WSN virus only provided partial protection against H3N2, with slight weight loss and 80% survival (Figure 5C), consistent with the viral load in the lungs of mice (Figure 5C). In contrast, the immunised group with either 10^2^PFU or 10^3^PFU PR8NARBD/WSN virus only provided partial protection against H7N9 virus challenge, with 60% and 80% survival, respectively. While 10^3^PFU PR8NAWSN group provided complete protection (Figure 5D). A higher viral load titre was detected in the lungs of the 10^2^PFU PR8NARBD/WSN group (Figure 5D). This indicates that the insertion of the RBD reduced protection against heterologous IAV challenge.

Histopathological analysis of lungs from challenged mice collected on day 5 dpi showed that the vaccinated group was largely protected against lung damage caused by H1N1 and H3N2 viral infection (Figure 5E–G). However, there was no significant difference in tissue damage in the vaccinated group with H7N9 challenge compared to the PBS group (Figure 5H). Taken together, these results indicate that a single dose of nasal immunisation with PR8NARBD/WSN can produce cross-protection against H1N1 and H3N2 IAV challenges but only partial protection against H7N9 viruses.

**Figure 6:**
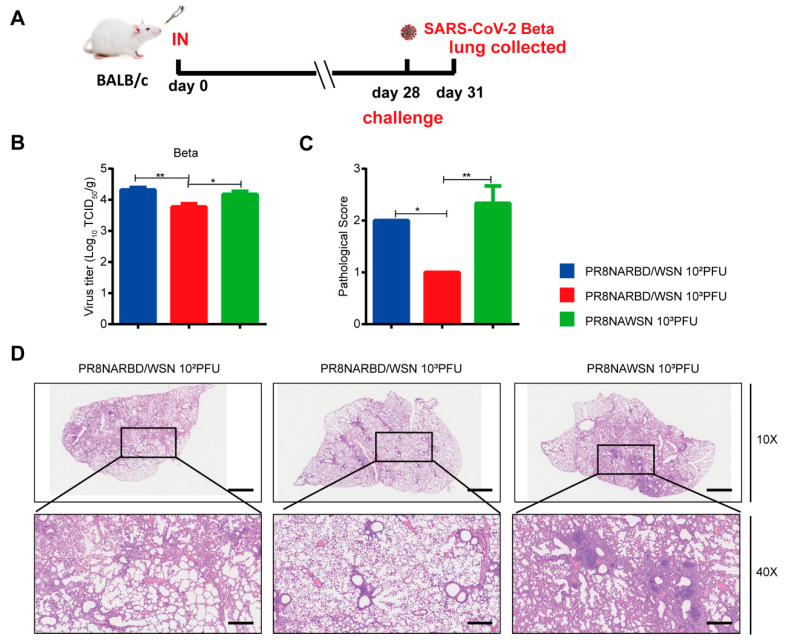
Protection against SARS-CoV-2 Beta viruses challenge in BALB/c mice through single intranasal immunisation with PR8NARBD/WSN virus. (**A**) Schematic of timeline for intranasal vaccination and SARS-CoV-2 Beta viruses challenge. (**B**) Virus titres in the lungs of BALB/c were measured at 4 dpi (*n* = 4 for each group). (**C**) Pathological score of mouse lung. (**D**) Representative images of H&E-stained lung sections. Scale bar, 100 μm. Statistical analysis was performed using one-way ANOVA. Error bars represent mean ± SEM (* *p* < 0.05, ** *p* < 0.01).

Mice immunisation with 10^3^PFU PR8NARBD/WSN viruses were protected against the lethal SARS-CoV-2 Beta challenge (Figure 6A). Lung viral loads in the 10^3^PFU PR8NARBD/WSN vaccinated group had a 2.9 times reduction compared to those in the PR8NAWSN group (Figure 6B). H&E staining of lung tissues further confirmed that 10^3^PFU PR8NARBD/WSN reduced pathological damage and inflammatory cell infiltration of blood vessels and bronchioles and minimised local alveolar wall breakage and alveolar cavity fusion (Figure 6C,D). These results demonstrated that a single dose of intranasal immunisation with PR8NARBD/WSN protects mice from heterogeneous SARS-CoV-2 Beta variant challenge.

## 4. Discussion

Both SARS-CoV-2 and influenza A viruses prefer the respiratory mucosal epithelium as a portal of entry and cause highly contagious respiratory diseases worldwide [1,27], highlighting the necessity to develop mucosal vaccines targeting both SARS-CoV-2 and IAV. In this study, we generated a novel bivalent mucosal vaccine, a PR8NARBD/WSN-based optimal chimeric H1N1 (WSN backbone), expressing the RBD of SARS-CoV-2.

Currently, inactivated COVID-19 vaccines, mRNA, and adenovirus vector vaccines, which are all intramuscular and do not produce mucosal immunity, play an important role in preventing severe COVID-19 illness or death, but are less effective in preventing infection and transmission. SARS-CoV-2 mucosal vaccines have been shown to be more effective than intramuscular injections in preventing infection and transmission by activating immune cells in mucosal tissues in the nasal cavity and respiratory tract [7,28,29]. Our data showed that a single dose of an intranasal immunisation with PR8NARBD/WSN produced mucosal immunity against SARS-CoV-2 and IAV and was found to be a promising bivalent vaccine for influenza and COVID-19.

It is well known that the annual seasonal IAV vaccine was developed using the backbone of A/Puerto Rico/8/1934 H1N1 (PR8) because of its attenuated phenotype in humans [30]. The A/WSN/33 H1N1 strains are no longer circulating among humans, and highly adapted laboratory strains, such as PR8, have been widely used to study live attenuated IAV (LAIV) vaccines [15,31,32]. Currently, the IAV H1N1-based recombinant COVID-19 vaccine (dNS1-RBD) has been approved for humans in China as an emergency use and a preliminary mechanistic study is also being conducted using the WSN backbone [31,33]. Recently, Loes et al. reported an IAV-vectored SARS-CoV-2 vaccine (∆NA (RBD) with a WSN backbone and an HA segment from the A/Aichi/2/1968 virus to decrease virulence. Single-dose intranasal inoculation of mice with ∆NA (RBD) virus resulted in the production of serum nAbs, but mucosal and cell immunity were not detected [15]. Moreover, a few reports have revealed that WSN are neurovirulent when inoculated intracerebrally [34], and this neurovirulence trait has been linked to NA in WSN [35]. In this study, we substituted the PR8 NA with WSN NA to ensure vaccine safety. Notably, IAV is a negative-segment RNA virus without a DNA intermediate, making it a safer vaccine delivery platform than most recombinant DNA viruses that can integrate their genes into host genomes. In general, PR8NARBD/WSN is a promising model for the development of a recombinant mucosal vaccine against other respiratory pathogens such as RSV.

The NA gene segment is a promising candidate for inserting exogenous genes, which can insert about 680 bp of exogenous genes without affecting the structure of NA [36]. The NA segment is a structural protein of IAV that is more likely to cause humoral and cellular immune responses in vivo. However, the low abundance of NA surface antigens is a bottleneck for improving the antibody responses to NA and foreign proteins [37]. Previous research has demonstrated that the wild-type WSN virus expresses more NA than the PR8 virus [16]. It is unclear whether the application of high NA expression in WSN is flexible for optimal IAV vector-based vaccines, particularly when the WSN NA gene is replaced by PR8NA. Here, we successfully verified that the seven WSN plasmids (HA, PB1, PB2, NP, PA, M, and NS) combined with PR8NA can restore higher expression of NA or its fragment-in-frame (such as the RBD of SARS-CoV-2 in this study) than the PR8 backbone. These results provide a foundation for the optimal design of NA-based IAV vector vaccines. Notably, the pathogenicity of PR8NARBD/WSN in mice was 35 times lower than that of PR8NARBD/PR8.

A combination of IFN-γ ELISpot assays and flow cytometry were used to characterise SARS-CoV-2 RBD-specific and influenza virus NP-specific T cells induced by PR8NARBD/WSN vaccination. The data indicated that the vaccine induced RBD-specific splenocytes and pneumonocytes IFN-γ T cell response. The expression level was higher than that previously reported for a prime-boost single cycle influenza virus-based SARS-CoV-2 vaccine (scPR8-RBD-M2) and comparable to the prime-boost level previously reported for the dNS1-RBD vaccine [7,12]. Flow cytometry assays showed similar results, with IFN-γ^+^ CD8^+^T cells detected after a single-dose immunisation.

Several recent experimental studies have reported the importance of memory T cells in the protection against respiratory viruses [25,38]. Trm is a key cell type that persists in tissues for a long time and helps limit mucosal pathogens and immune sentinels. It also provides rapid and broad-spectrum protective effects against a variety of respiratory infection from previously encountered pathogens. The past experiences of specific memory T cells persisted in SARS-recovered patients for up to 6 years post-infection [39], indicating that Trms are essential to produce long-term containment of SARS-CoV-2 virus in the next generation of COVID-19 vaccine. SARS-CoV-2 vaccination induces immunological T cell memory able to cross-recognise variants from Alpha to Omicron [23]. Recent studies have also demonstrated the endurance of the SARS-CoV-2-targeted memory T cell response, which can be present for up to 10 months [40]. Furthermore, several studies of cells from human lung donors have identified influenza-specific Trms that are cross-reactive against multiple influenza strains. Trms are found in the lower (lung) respiratory tract, and these cells play a critical role in local defence against respiratory infections [41]. The co-expression of CD69 and CD103 can be used to identify CD8^+^ Trms in the lungs of both mice and humans [41], and our data suggest that a single dose of intranasal immunisation with PR8NARBD/WSN produces Trm cells against SARS-CoV-2 and IAV. The RBD- and NP-specific CD69^+^CD103^+^ T cell expression levels are similar to those prime-boost level reported previously for the dNS1-RBD vaccine and LAIV-RSV vaccines [7,42].

This study has some major limitations. First, only the prototype RBD of SARS-CoV-2 inserted in the NA segment was tested; no other targets were studied (for example, the omicron RBD of SARS-CoV-2 or peptides of cross-protective T cell epitopes against variants of concern of SARS-CoV-2). Second, we only tested this recombinant vaccine in mice and did not assess its immunogenicity and protection in other animal models. Third, we only detected immunogenicity in IAV-native mice. Previous studies have shown that pre-existing immunity against vaccine vectors can reduce vaccine effectiveness [43]. These limitations may be overcome by substituting the HA subtypes.

In conclusion, our current data provide evidence that PR8NARBD/WSN is a unique mucosal vaccine candidate for single-dose intranasal immunisation that provides broad protection against IAV and SARS-CoV-2. Our research demonstrates that PR8NARBD/WSN is an attractive vaccine target for emerging SARS-CoV-2 variants and IAV infections. Continued work on this vaccine platform will help in present and future responses to pandemic respiratory pathogen infections.

## Data Availability

The datasets generated during the current study are available from the corresponding author on reasonable request.

## Supplementary Materials

The following supporting information can be downloaded at https://www.mdpi.com/article/10.3390/vaccines11091453/s1, Methods S1.1 Genetic stability of recombinant virus and growth kinetic. Methods S1.2. Western blot. Methods S1.3. Neuraminidase activity measurements.Methods S1.4 RBD concentration was detected by Enzyme-linked immunosorbent assays (ELISAs). Methods S1.5. SARS-COV-2 Pseudotypes Virus Neutralization Assay. Methods S1.6 Hemagglutination Inhibition Assays (HAI). Figure S1: Humoral immune and cellular immune responses elicited by single-dose intranasal immune PR8NARBD/PR8 viruses in BALB/c mice. Figure S2: Gating strategy for memory T cell phenotypes. Exemplary gating strategy of a lung sample from a PR8NARBD/WSN immunised mouse.
