# Supplementary material for: Single-Dose Intranasal Immunisation with Novel Chimeric H1N1 Expressing the Receptor-Binding Domain of SARS-CoV-2 Induces Robust Mucosal Immunity, Tissue-Resident Memory T Cells, and Heterologous Protection in Mice
Source: Vaccines (Basel). 2023 Sep 4;11(9):1453. doi: 10.3390/vaccines11091453 (PMC10537001; doi:10.3390/vaccines11091453)
Supplement: Supplementary file 1 [file vaccines-11-01453-s001.zip › vaccines-2556045-supplementary.pdf]

vaccines

MDPI

Supplementary material

Single-dose intranasal immunisation with novel chimeric H1N1 expressing the receptor-binding domain of SARS-CoV-2 induces robust mucosal immunity, tissue-resident memory T cells, and heterologous protection in mice

Donghong Wang [1], Yao Deng [1], Jianfang Zhou [2], Wen Wang [1], Baoying Huang [1], Wenling Wang [1], Lan Wei [1], Jiao Ren [1], Ruiwen Han [1,3], Jialuo Bing [1,4], Chengcheng Zhai [1], Xiaoyan Guo [1] and Wenjie Tan [1,3,4,*]

1 Key Laboratory of Biosafety, National Health Commissions, National Institute for Viral Disease Control and Prevention, China CDC, 155 Changbai Road, Beijing 102206, China
2 State Key Laboratory for Molecular Virology and Genetic Engineering, Chinese National Influenza Center, National Institute for Viral Disease Control and Prevention, China CDC, 155 Changbai Road, Beijing 102206, China
3 Zhejiang Provincial Key Laboratory of Medical Genetics, School of Laboratory Medicine and Life Sciences, Wenzhou Medical University, Wenzhou 325035, China
4 School of Public Health, Xinxiang Medical University, Xinxiang 453003, China
* Correspondence: tanwj@ivdc.chinacdc.cn

1.Supplemental Methods

1.1 Genetic stability of recombinant virus and growth kinetic

The viruses of PR8NARBD/PR8,PR8NARBD/WSN were propagated in 9-day-old chicken eggs purchased from Merial, at 100 PFU of each virus was injected into egg. After incubation at 37℃ for 48 h, the virus allantoic fluid was harvested and titrated for TCID50 values. In order to test the genetic stability of recombinant virus, the virus was serially passaged in 9-day-old embryonated eggs five times and collect the allantoic fluid, labeled as E1 to E5 accordingly. The viruses of passage E1 to E5 were extracted using QIAamp Vira RNA Mini Kit (REF:52906) according to the manufacturer's instruction and converted to cDNA by Superscript® III First- strand kit (Invitrogen).The unit12 primer (5′-AGCA/GAAAGCAGG-3′) were used to amplified the full-genome with QIAGEN One-step RT-PCR kit and visualized by 1% agarose gel electrophoresis. The PR8-NA-RBD specific fragments were amplified with NA universal primer Ba-NA-1/Ba-NA-1413R described by E.Hoffmann[1].The sequences was further confirmed by Sanger sequencing.

1.2 Western blot

The virus allantoic fluid was collected and concentrated purification as previously described[2]. Membranes were probed with rabbit anti-Spike RBD (Biodragon, cat:BD-PD400014), mouse anti-influenza A NP (Millipore, MAB 8257) , Goat anti-rabbit IgG-HRP (KPL,MA, USA) and anti-mouse IgG-HRP antibodies were used as secondary antibodies for chemiluminescence detection by AEC substrate set(551951) and imaged using chemiluminescent imager.

1.3 Neuraminidase activity measurements

NA activity was measured by NA-Fluor™ Influenza Neuraminidase Assay Kit. The viruses were performed serially two-fold dilution across the black plate in 1xNA-Fluor buffer. Add 50 μL of 200 μM MUNANA per well and gently tap the plate to mix. Incubate the plate at 37 °C for 1 h. Add 100 μL of stop solution per well to terminate the reaction and gently tap the plate to mix. Read the plate using a fluorometer.

1.4 RBD concentration was detected by Enzyme-linked immunosorbent assays (ELISAs)

The RBD concentration of the reassortment viruses PR8NARBD/PR8 and PR8NARBD/WSN was quantitative by enzyme-linked immunosorbent assay (ELISA) using SARS-COV-2 Spike RBD kit (Sino biological, Cat:KIT40592). It contains recombinant SARS-CoV-2 Coronavirus spike, and antibodies raised against the recombinant RBD protein. Briefly, Prepare serially diluted standard to quantified the concentration of the virus stock. The viruses samples were diluted to the right concentration and does not exceed the standard curve and 100μL of virus sample were added to per well, Incubate for 2 hours at room temperature.Add 100μL of Detection Antibody in working concentration to each well. and incubate for 1 hour at room temperature.Reaction were stopped with stop soulation and read at 450nm.

1.5 SARS-COV-2 Pseudotypes Virus Neutralization Assay

Neutralizing antibodies against SARS-COV-2 in serum were detected by Pseudotypes Virus Neutralization Assay based on previous study[3]. Pseudotyped SARS-CoV-2 virus with the Fluc reporter gene were generated and titrated as described previously[3]. Mouse sera were heated inactivated at 56°C for 30 min and serially 2-fold diluted (starting dilution of (1:20) in 2%FBS in DMEM. Huh7.5 cells were inoculated in a 96-well cell culture plate and 80% confluency.The diluted serum was mixed with equal volume of pseudovirus particles, and the mixture was added into Huh7.5 cells incubated at 37°C for 2h. Cell were washed and overlaid with DMEM containing 2% FBS and incubated at 37°C48 h, the Bright-Glo luciferase assay substrate was added to each well, and the luminescence was measured with GLOMAX luminometer, and the neutralization efficiency was then calculated.

1.6 Hemagglutination Inhibition Assays (HAI)

To evaluate neutralization antibody against Influenza viruses,the HAI assay were performed as follows. Serum samples from mice were treated with receptor-destroying enzyme (RDE (II); Denka Seiken Co., Ltd, cat # 370013) for 18h at 37 °C and heat inactivated for 30 min at 56 °C. Then, the two-fold serially diluted serum samples were mixed with the PR8NAWSN virues(4 hemagglutinating units,4HAU) and culture 30 min at RT. HAI titers were determined by adding 0.5% turkey red blood cells to the virus-Ab mixtures followed by a 30 min incubation at RT. the HAI titers were defined as the reciprocal of the highest serum dilution for complete hemagglutination inhibition.

2. Supplemental Figure

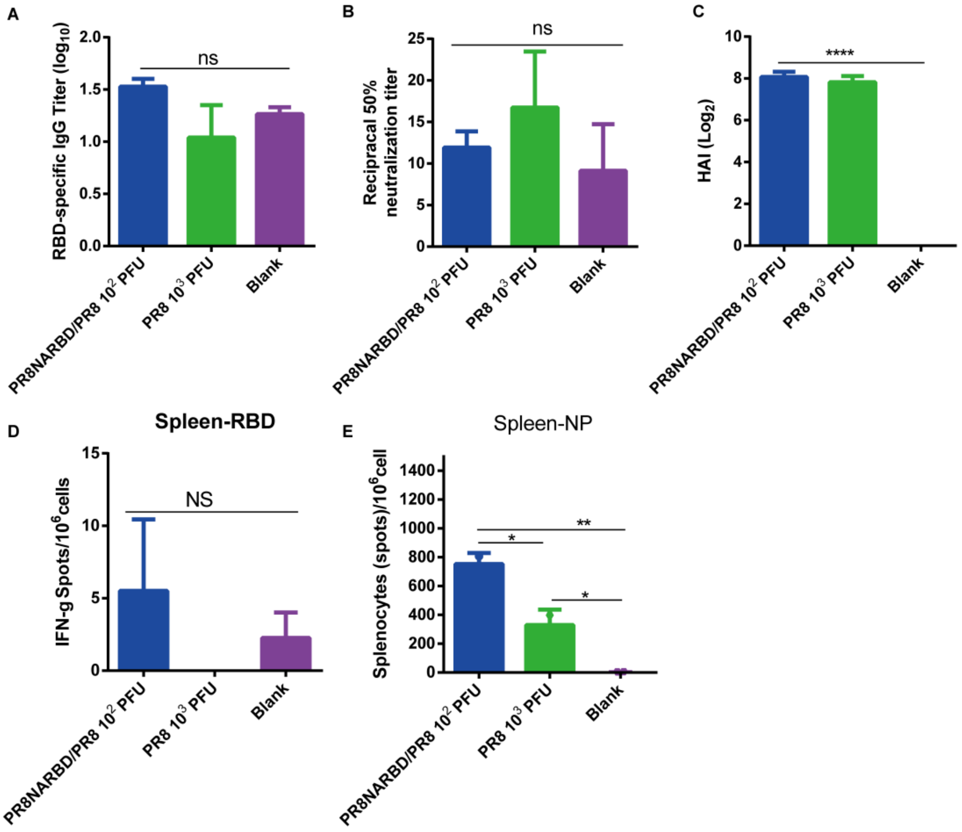

Supplemental Figure S1: Humoral immune and cellular immune responses elicited by single dose intranasal immune PR8NARBD/PR8 viruses in BALB/c mice. Serum and Splenocytes were obtained on day 14 after a single intranasal administration of PR8NARBD/PR8,(A) RBD specific binding antibody (B) RBD specific neutralization antibody. (C)Serum HAI titers against the PR8 virus. (D)RBD specific cell immunity. (G) NP specific cell immunity.Statistical significances were analyzed by one-way analysis of variance (ANOVA).The bars plotted show means±SEM. The results represent three independent experiments. (*p < 0.05, **p < 0.01, ***p < 0.001, ****p <0.0001).

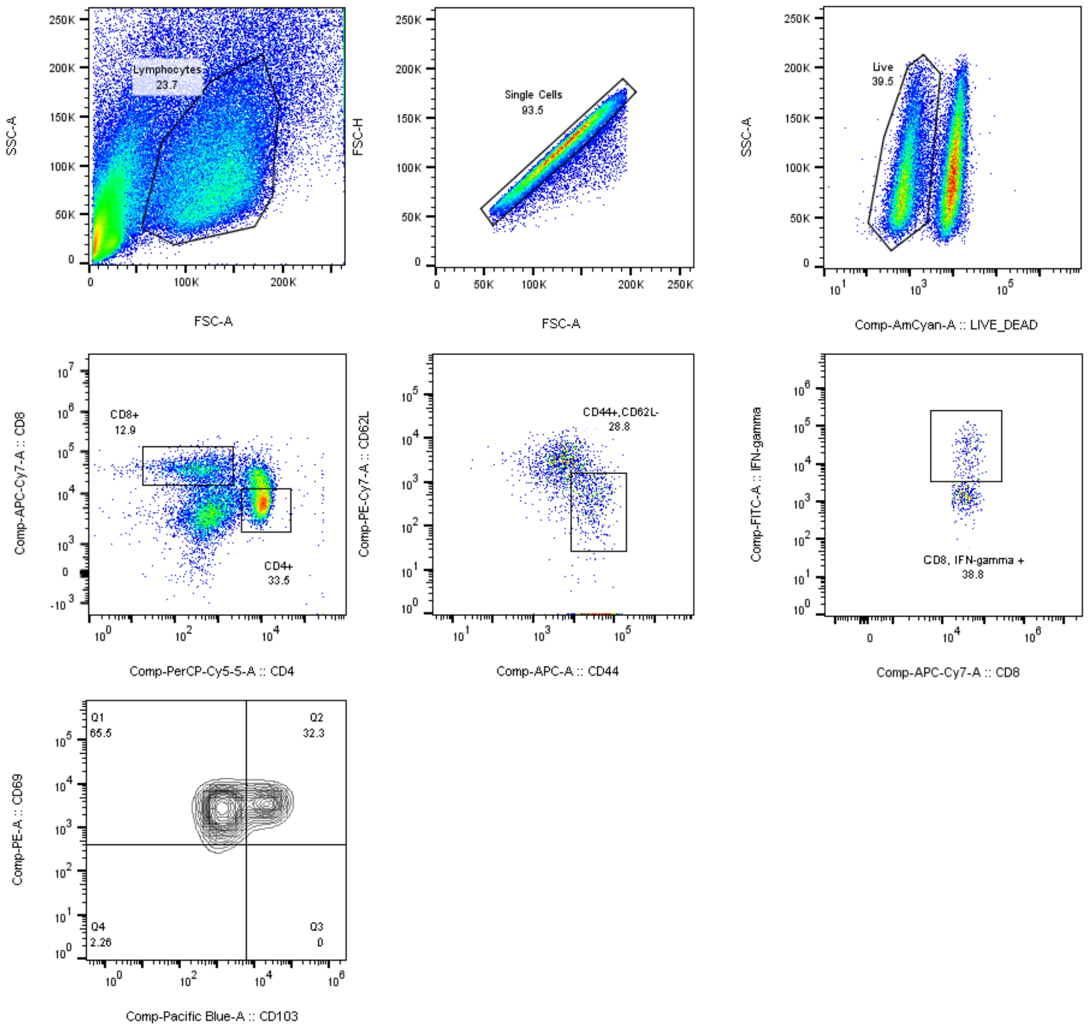

Supplemental Figure S2: Gating strategy for memory T cell phenotypes.Exemplary gating strategy of a lung sample from an PR8NARBD/WSN immunized mouse.lung lymphocytes were collected 14 days later and the expression of memory pneumonocytes was detected ($CD8^{+}CD44^{+}CD62L^{-}$ $CD69^{+}CD103^{+}$) with NP peptides.

Supplemental References

1. Hoffmann E, Krauss S, Perez D, et al. Eight-plasmid system for rapid generation of influenza virus vaccines. Vaccine. 2002 Aug 19;20(25-26):3165-70.
2. Gao J, Wan H, Li X, et al. Balancing the influenza neuraminidase and hemagglutinin responses by exchanging the vaccine virus backbone. PLoS Pathog. 2021 Apr;17(4):e1009171.
3. Yang R, Huang B, A R, et al. Development and effectiveness of pseudotyped SARS-CoV-2 system as determined by neutralizing efficiency and entry inhibition test in vitro. Biosaf Health. 2020 Dec;2(4):226-231.
